# In-Process Error-Matching Measurement and Compensation Method for Complex Mating

**DOI:** 10.3390/s21227660

**Published:** 2021-11-18

**Authors:** Shih-Ming Wang, Ren-Qi Tu, Hariyanto Gunawan

**Affiliations:** 1 Department of Mechanical Engineering, National Chung Hsing University, Taichung 40227, Taiwan; shihmingbear@nchu.edu.tw; 2 Department of Mechanical Engineering, Chung Yuan Christian University, Taoyuan 320314, Taiwan; mango2309@yahoo.com.tw; 3 R&D Center for Smart Manufacturing, Chung Yuan Christian University, Taoyuan 320314, Taiwan

**Keywords:** error mating, complex mating, in-process measurement, error compensation

## Abstract

This study proposed an error-matching measurement and compensation method for curve mating and complex mating. With use of polynomial curve fitting and least squares methods for error analysis, an algorithm for error identification and error compensation were proposed. Furthermore, based on the proposed method, an online error-matching compensation system with an autorevising function module for autogenerating an error-compensated NC program for machining was built. Experimental verification results showed that the proposed method can effectively improve the accuracy of assembly matching. In a curve-type mating experiment, the matching error without compensation was 0.116 mm, and it decreased to 0.048 mm after compensation. The assembly accuracy was improved by 28%. In a complex-type mating experiment, the verification results showed that the error reductions after compensation for three mating shapes (straight line, triangle, and curve shape) were 81%, 87%, and 79%, respectively. It showed that the proposed method can improve the assembly accuracy for complex mating shapes, which would also be improved without losing production efficiency.

## 1. Introduction

In response to the needs of manufacturing applications, the international industry, academia, and research institution sustain the research on improving the machining accuracy of the machine through hardware improvement or software-based error compensation. For production lines with high-precision requirements, due to the tight tolerance zone design of each process, high-specification and high-precision machines are usually required for each process, resulting in higher hardware costs for the production line. Therefore, if the real-time error measurement and compensation in the manufacturing process enable the front and back processes to perform matching error compensation processing, then the matching between the processes can be greatly improved and the re-work rate and defect rate can be reduced, thereby reducing the manufacturing cost of the production line.

In addition, in the production of precision industrial products, it is often necessary to individually manufacture components and then perform the precision assembly. The assembly matching accuracy depends on the requirements of individual processing accuracy and the selection of two components (part and counterpart) with the smallest matching error. At present, most of the production methods in the industry are mass production, and then suitable part and counterpart are manually matched and selected for assembly. However, due to the different manufacturing processes of the parts and the counterparts, and the different manufacturing tolerance ranges, the cost of maintaining manufacturing accuracy is high; additionally, the manual selection is time-consuming and inefficient. The online curve shape measurement and matching error compensation system proposed in this research is to parallel and efficiently measure the error of the part on the production line, and then convert it into the correction error of the counterpart to be machined, afterward automatically generate the numerical control (NC) processing program with the correction. With high-efficiency information communication, the suitable counterpart is machined, so that the matching accuracy between the part and its counterpart is improved in the low-cost and high-efficiency manufacturing process. Another advantage of this method is that there is no need to require strict tolerances in every manufacturing process, which makes the production line more robust and results in lower production costs.

The matching shapes between the part and its counterpart can be divided into straight, triangle, and curve types. Our previous study proposed matching error compensation methods for straight and triangle matching [1,2]. This research proposes more complex matching shapes that are curved matching error measurement and compensation methods to meet the industry’s demand for complex assembly matching. The proposed method can be used for automated production lines with CNC machine tools, robotic arms, and measurement systems. The autoerror compensation module and production line monitoring module can be integrated with the automation production line to improve production line efficiency, and greatly reduce unnecessary manual matching costs. The compensation method used in the research is passive compensation. By measuring the positional coordinates of the mating area of the part, the deviation and geometric error of the mating area are automatically calculated, and then the measured error is converted into a correction amount for its counterpart through the error conversion module. Subsequently, use the established NC program autoidentification and compensation module to compensate the correction amount to the corresponding section of the NC program, and finally use the compensated NC program to machining the counterpart, thus the counterpart can be accurately matched with the part.

In the past, many studies have investigated error compensation methods to improve the machining accuracy of the machine. Wang et al. [3] proposed an on-machine and vision-based measurement method to measure the volume error of a micromachine tool then compensated to the machining trajectory. Zhang et al. [4] used Cross Grid Encoder KGM181 to measure and identify the geometric error and developed an automatic compensation system module. Jia et al. [5] comprehensively summarized and classified contouring-error reduction methods for three-axis and five-axis CNC. The advantages and disadvantages of different kinds of methods were discussed and compared. Yang et al. [6] improved the tracking accuracy of the CNC machine tool by establishing a two-stage feedforward friction compensation model. Experimental verification was also conducted and showed that the proposed method could improve the tracking accuracy of CNC machine tools by around 20%. Wan et al. [7] proposed a three-axis CNC geometric error model using a homogeneous transformation matrix, which converts high-order nonlinear spatial geometric problems into algebraic equations by fitting geometric error components through a cubic polynomial function. Nghiep et al. [8] investigated the mechanism of tool deflection error, then minimized the deflection error by controlling the cutting parameters and suitable lubrication mode. Zhao et al. [9] proposed an effective error compensation method with a new error prediction model and error compensation strategy for coordinated five-axis machine tools. The influence of the coordinated workspace of prismatic joints (CWP) and the coordinated workspace of revolute joints (CWR) on the objective error was investigated, then it was combined with the interpolation algorithm to predict the relative position and orientation error. Zhou et al. [10] investigated nonlinearity error on five-axis CNC machining. A close-form representation of the envelope surface methods was proposed to calculated the nonlinearity error. The Cutter location data from commercial computer aided manufacturing (CAM) and practical machining experiment were collected and compared to determine the surface deviation. Shi et al. [11] investigated the degradation of contouring accuracy of machine tools caused by transient backlash error (TBE). A mathematical model for feed drive with backlash was developed from full-closed loop type feed drive. Based on this model, the TBE was demonstrated. Simulation and experiment were carried out for validation, and it showed the magnitude of TBE could be forecasted if the open-loop gains and the backlash widths are identified. Bi et al. [12] proposed an adaptive machining method for curved contour based on a novel isometric mapping. The method includes three steps: the first, using a laser scanner based on-machine measurement (OMM) system to obtain the real geometry of the deformed surface. The second, establish an isometric mapping between two sets of points. The accuracy of matching between the nominal surface and the actual surface is defined according to the deviation of the geodesic distance of the two sets of points. The third adaptively adjusts the toolpath according to the result of isometric surface mapping to compensate for the deformation error. Simulation and machining experiments were carried out to prove the feasibility and effectiveness of the proposed method. Huang et al. [13] developed a new elastic deformation compensation interpolation (MEDCI) algorithm to generate a modified position command, thereby reducing the tracking error caused by elastic deformation.

Monitoring and control of the manufacturing process are important for the development of manufacturing industries. Process monitoring is the manipulation of sensor measurement (e.g., vibration, force, temperature) in determining the state of processes. Different real-time monitoring techniques to monitor the manufacturing process have been investigated, such as Dinardo et al. [14] used vibration signals to continuously monitoring the machine condition. This approached allows for self-assessment of health and degradation status of the machine system. Teti et al. [15] provided a comprehensive review of sensor technology, signal processing, and decision-making strategies for machining monitoring. Different techniques and methods of signal feature extraction and feature integration for decision making were elaborated and discussed. Han et al. [16] proposed a novel method to predict health management of complex multi-state manufacturing systems. The hard failure and soft failure of the manufacturing system were defined. With considering the functional dependence of the manufacturing component and soft failure, developed Remaining Useful Life (RUL) method. The functional importance of the manufacturing component was defined and used for optimizing the process of manufacturing system maintenance decision making.

For high-precision products, measurement accuracy plays a key role. The Coordinate Measuring Machine (CMM) is widely used in industry to evaluate dimensional and geometric characteristics of complex high precision parts. However, due to the demand for shorter cycle times of measurement tasks, in such conditions, dynamic errors will certainly have a much more influence on the measurement accuracy. Echerfaoui et al. [17] investigated the dynamic errors in the CMM through experimental work. Experimental design and statistical analysis tools were combined to evaluate the measurement parameters effects at high measuring velocity, then these parameters were used to investigate the variation of dynamic error. Ostrowska et al. [18] developed a virtual articulated arm coordinate measuring machine (VAACMM) using three different metrological models. Verification method based on measurements of multi-feature check standard and posterior predictive p-value test was proposed for assuring the correct functioning of developed VAACMM. Xing [19] et al. proposed a method that combined volumetric errors (VEs), vector similarity measures (VSMs), and the exponentially weighted moving average (EMWA) for machine tool accuracy monitoring. Simulation machine error data and the real machine tool test were carried out to verified the proposed method. The results showed that VE is significant for monitoring the machine tool accuracy condition, and VSMs work well in VE feature extraction. Yang et al. [20] developed a high-accuracy online prediction algorithm of five-axis contouring errors according to three-point arc approximation (TPAA). The experiment results showed that the proposed TPAA algorithm can predict contouring error with higher accuracy than linear segment approximation (LSA). Tang et al. [21] approached a new analyzing method to calculate the straightness and angular errors according to measuring guideway surface and fitting curve. Jia et al. [22] proposed the Non-Uniform Rational B-Splines (NURBS) interpolator method for the calculation of the contour error. A parameter compensation-based second-order Runge–Kutta method was used to precisely calculate the new interpolation-point parameter. Mao et al. [23] proposed a new method based on resampling to create surface fitting for the data from the Coordinate Measuring System. The method consists of three parts: NURBS curve fitting for each row, resampling on the fitted curve, and surface fitting from the resampled data. The numerical experiments were conducted with simulation and practical data. The results demonstrated fast, effective, and robust calculation.

The previous research on error compensation mostly focused on the improvement of the processing accuracy of a single part and less focused on the assembly parts. This research focuses on the measurement and error compensation for high-precision assembly matching. The error of a part is measured in real time on the production line, then the error compensation value for its counterpart is calculated according to the error of the part. Furthermore, the error compensation value is compensated into the counterpart NC program. Finally, the counterpart is machined using a compensated NC program. Thereby, if an error exists on a part, the error is autocompensated to its counterpart to be machined, so that the high-precision matching assembly can be obtained. In the past, our research team has proposed compensation methods for straight and triangle matching errors [1]. This paper extends a method for measuring and error compensation for curved and complex matching errors. In Section 2, the matching error measurement and compensation methods were described and elaborated. Section 3 described the online error measurement and compensation system developed based on the proposed method. In Section 4, the experiments and verification results were presented and discussed. Finally, the conclusion was summarized in Section 5.

## 2. Methodology

In the matching error measurement and compensation method, the user needs to define the key nodes in the mating area of a part, then the coordinate of the key nodes is directly measured on the production line to create the actual size and geometric profile. The tolerance chain in the design was included in the calculation of the theoretical minimum and maximum dimensions of the part. Subsequently, the size and geometric profile obtained from the measured part are compared with the theoretical size and geometric profile to determine the size and profile error of the part, and then convert into the compensation value of the counterpart to be machined. Finally, a corrected counterpart NC program is autogenerated according to the compensation value, then machine the counterpart that matching to the part. The methodology includes: (1) the selection of the key nodes, (2) measurement methods, (3) curved contour reconstruction, (4) error comparison, and (5) error conversion and NC program autocorrection.

### 2.1. Selection of Key Nodes

Before calculating the error and compensation, the product needs to be measured by the measurement instrument. First, it is necessary to define the key measurement points in the part mating area, so that the complete measurement information of the actual size and contour profile can be obtained and reconstructed. There are three types of matching assembly shapes between the part and counterpart, namely straight line, triangle, and curved. The straight line matching focuses on straight and length. To construct a straight line, two points are needed. The triangle matching focuses on the triangle side length and the angle. To construct a triangle, it is necessary to create two lines and the angle between the line must be known. Therefore, four points are needed to create two lines and using dot product equation to obtain the length of two lines and the angle between the lines. For the curve shape, the required points to construct a hill or valley are at least three points. However, for large hills or valleys, it is recommended to add one point for every increase of 5 mm. To construct the curve shape, the polynomial regression and least squares methods were used. The number of points will influence the accuracy of the curve construction. More number of points will provide higher accuracy of curve reconstruction, but it will take a longer measurement time. Alternatively, a fewer number of points will result in lower accuracy of curve reconstruction, but shorter measurement time. The mating error of the part can be obtained by comparing the actual shape after machining and the theoretical shape from the CAD. Furthermore, this mating error value is used to modify the shape of its counterpart to obtain precision matching in assembly.

### 2.2. Measurement Method

**Figure 1:**
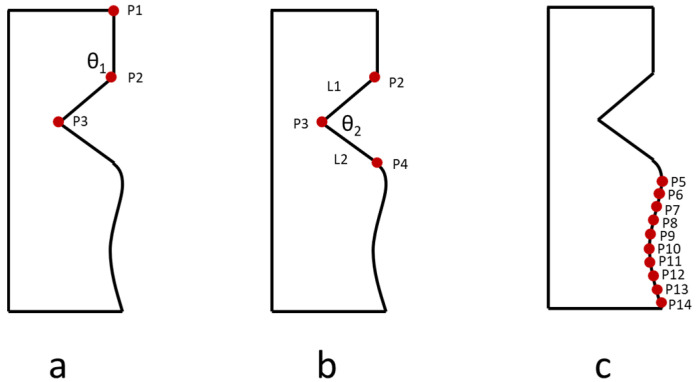
Illustration of the mating area that consists of straight line, triangle, and curve shapes. (**a**) keypoints to measure straight line, (**b**) keypoints to measure triangle, and (**c**) keypoints to measure curve.

The point-to-point measurement method in the Renishaw Equator 300 is used to measure the part. This method uses several points to reconstruct the shape of the part. First, the origin position of the machining coordinate system must be checked, then the coordinates of the key points must be measured according to this coordinate system; furthermore, the actual curve must be reconstructed based on the measured coordinates. Subsequently, the actual curved and theoretical curved (from CAD drawing) is compared, and then the deviation is calculated between the theoretical point and the actual point. Figure 1 shows the mating area that consists of a straight line, triangle, and curve shape. The two key measurement points of the straight line are the point P1 and P2 in Figure 1a. The coordinates of P1 and P2 can be directly measured by the Renishaw Equator 300 measurement instrument. The length of the straight line can be obtained through these two points. For the angle of the triangle shape, the angle between the two lines can be calculated using a dot product, for example, $\theta_{2}$ can be calculated using Equation (1).

(1) $$\theta_{2}=\cos^{-1}\left(\frac{\vec{L1}\cdot \vec{L2}}{\left\|\vec{L1}\|\cdot \|\vec{L2}\right\|}\right)\;$$

For the curve shape, the point-to-point measurement method is used instead of the scanning measurement method. The advantage of using the point-to-point method is that it only needs several points to construct the curve, and the measurement time is short compared with the scanning method that uses numerous points and a longer measurement time. However, the number of points will affect the accuracy of the curve reconstruction. More points will produce higher accuracy of curve shape but longer measurement time is required as a consequence; on the other hand, fewer points will result in lower accuracy, but the measurement time is shorter. For example, ten points were taken while measuring the curve, as shown in Figure 1c.

As is known, the measurement has some degree of errors that may come from the instrument or loading/unloading. The Renishaw Equator 300 that used in this study is an automated flexible gauge that employs the comparator principle via RenCompare software to account for the influence of systematic effects associated with Coordinate Measurement System (CMS) [24,25]. The parallel kinematic-based mechanism is used in the Equator instrument to minimize the machine’s dynamic errors at high measurement speed. To handle the temperature effect of the environment, the re-mastering process can be managed with the built-in sensor and software configuration [26]. Accordingly, in comparative coordinate measurements, the main uncertainty contributors are environmental effect, machine repeatability, calibrated master part, part fixture, sampling strategy, and the geometric element best-fit algorithm. The critical factor that affects to coordinate measurement machine is the part-alignment procedure used to define the coordinate system or frame of reference. In coordinate measurement, an improper part fixture set-up influences the measurement accuracy. When each part is fixed within 1 mm relative to the master part, size and position measurements made immediately following re-mastering may have a comparison uncertainty of ±2 μm relative to the certified measurement of the master part. Angular misalignment can be avoided by using an appropriate fixture for part holding. However, clamping the part to be inspected in the proper position and orientation is not always easy. To minimize the loading/unloading errors, the Erowa Power Chuck tooling system with repeatability <5 μm was used to hold the part.

The uncertainty of Equator comparator measurement for touch-trigger probing (TTP) mode is less than 0.6 μm. The number of probing points has no significant influence on the comparator measurement uncertainty, and comparison uncertainty is less than 0.5 μm for the angular misalignment within a range of ±1 mm. A large number of contact points provides smaller measurement uncertainty. The length measurement uncertainty of the Equator comparator remains below ±2 μm when the fixture/component is relocated within an error range of ±1 mm (fixture requirement is according to the Equator system specification) [1].

**Figure 2:**
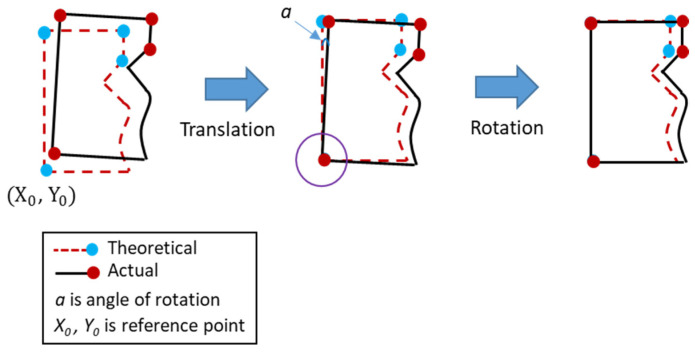
The translation and rotation of the measurement coordinates.

For the part-alignment in the measurement, the coordinate translation and rotation conversion as shown in Figure 2 were performed. The rotation conversion formula can be expressed as Equation (2).

(2) $$\{\begin{array}{c} X_{2}=\left(X-X_{0}\right)*\cos a-\left(Y-Y_{0}\right)*\sin a+X_{0}\\ Y_{2}=\left(X-X_{0}\right)*\sin a-\left(Y-Y_{0}\right)*\cos a+X_{0} \end{array}$$

where $X_{2}$, $Y_{2}$ are coordinate after rotation; X, Y are coordinate before rotation; $X_{0}$, $Y_{0}$ are center of rotation (reference point).

### 2.3. Fitting Method

**Figure 3:**
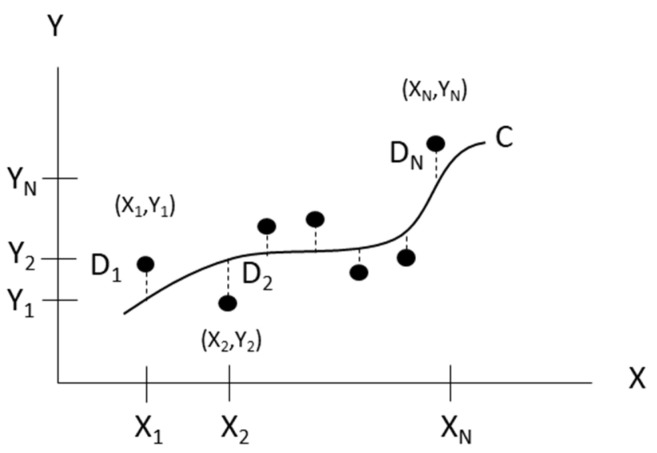
Schematic diagram of curve fitting.

The straight line fitting and geometric angle fitting are common problems in engineering applications. The least squares (LS) or total least squares (TLS) method can be used to fit the two-dimensional straight line. However, the LS or TLS method cannot be directly used to fit the three-dimensional spatial straight line due to the large number of equations to be solved; the solving process is relatively cumbersome, with poor practicability. Therefore, the three-dimensional problem is converted into a two-dimensional problem, then LS or TLS methods are used to fit the straight line. Polynomial curve fitting is a common method of data fitting. Polynomials can be used to fit data points in two ways. First, the polynomial passes through all the data points, and second, the polynomial does not necessarily pass through any of the points, but gives a good approximation of the data overall. Figure 3 illustrates N data points with their corresponding coordinates (X_1_, Y_1_), (X_2_, Y_2_)… (X_N_, Y_N_) and curve-fitted line. For a given coordinate, such as (X_i_, Y_i_), a deviation D_i_ exists between the corresponding value on the Y-axis and the Y value on the curve C. This deviation value can be positive, negative, or zero. The maximum order of the polynomial is dictated by the number of data points used to generate it, and can be calculated as m = N − 1. The polynomial can be created passing through all the points when the polynomial of the degree is N − 1. On the other hand, if the polynomial of the degree is less than N − 1, the polynomial does not pass through any of the points, but overall approximates the data. The general form of polynomials function can be written as Equation (3).

(3) $$f\left(x\right)=a_{n}x^{n}+a_{n-1}x^{n-1}+\cdots +a_{1}x+a_{0}\;$$

where $a_{n},a_{n-1},\ldots ,a_{1},a_{0}$ are the coefficients, and *n* is the order of the polynomials.

The higher degree polynomials may give a larger error or are more likely to overfit. To obtain the best fit curve line to data points, the least squares method was used to minimize the sum of the squares of the residuals at all the data points. In addition, to avoid overfitting problems in curve construction using the polynomials method, regularization was used in the least squares to control the overfitting. The smaller the value, the better the fitting degree. The minimum sum of squares of error can be calculated using Equation (4).

(4) $$Q_{L}=\overset{N}{\underset{i=1}{\sum }}{\left[y_{i}-\left(ax_{i}+b\right)\right]}^{\;2}\;$$

The polynomial curve fitting with the minimum sum of squares of error can be calculated using Equation (5).

(5) $$Q_{c}=\overset{N}{\underset{i=0}{\sum }}{\left(y_{i}-\left(a_{0}+\overset{m}{\underset{j=1}{\sum }}a_{j}x^{j}\right)\right)}^{2}\;$$

### 2.4. Error Comparison

**Figure 4:**
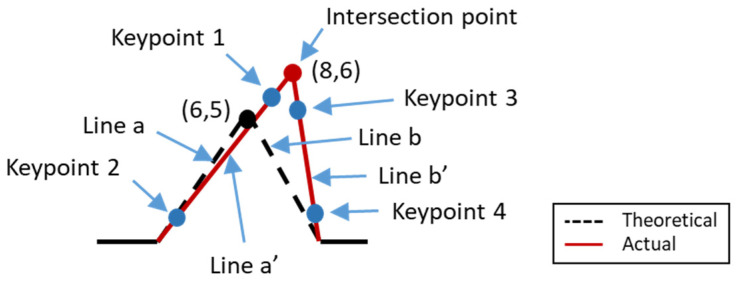
Illustration diagram of compensation.

The theoretical coordinate of the key points of the workpiece can be obtained from the CAD drawing, whereas the actual coordinates of the workpiece can be obtained by measuring the machined workpiece. For instance, Figure 4 illustrates the theoretical shape (dashed line) of a part. Assuming that the theoretical coordinates of the pick point are (6,5), then this coordinate value will be entered into the proposed error compensation system setting. After machined the part, the actual shape of the part was measured by using Renishaw Equator 300 instrument. The measurement probe will touch two points (keypoint 1 and 2) on the side a and (keypoint 3 and 4) on side b. Subsequently, straight line a’ and line b’ were constructed according to those points, and the intersection point between straight lines a’ and b’, which is (8,6), was obtained, as shown in Figure 4. The error is determined by comparing between actual part coordinate value and theoretical part coordinate value. In addition, the tolerance chain is also included in the error comparison. The maximum and minimum dimension of part is calculated according to the tolerance chain. For the error of part in the tolerance chain, it does not need to compensate to the counterpart. On the other hand, if the error of the part exceeds the tolerance chain, it is needed to compensate the error to the counterpart.

### 2.5. Error Conversion

The actual measurement information is provided in CSV format. Therefore, a CSV data-parsing module was established to analyze the content of the source file and determine whether the data content belongs to the part or counterpart.

**Figure 5:**
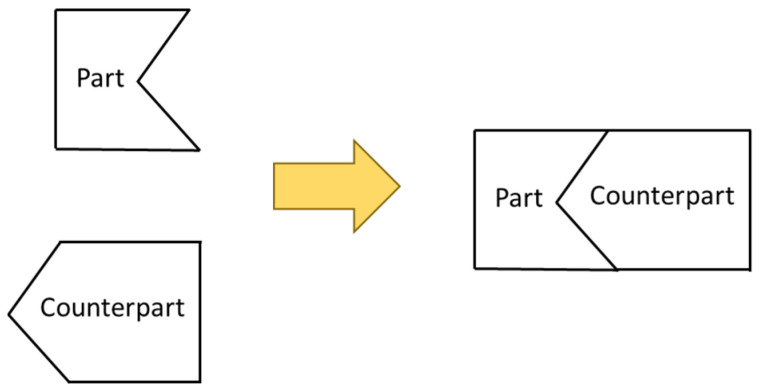
Illustration of part and counterpart.

**Figure 6:**
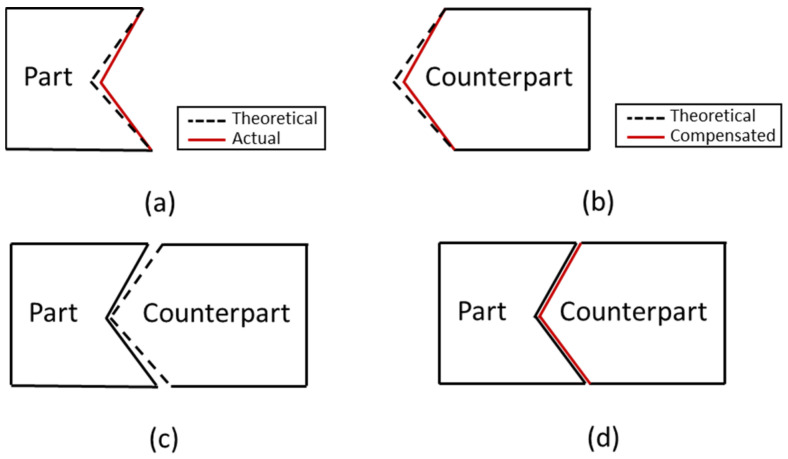
(**a**) Part shape, (**b**) counterpart shape, (**c**) assembly matching without compensation, (**d**) assembly matching with compensation.

To easily understand the error conversion, an illustration assembly between part and counterpart is depicted in Figure 5. To convert the part error to the counterpart error, the first step is to measure the actual shape of the part, as shown in the red solid line in Figure 6a, then calculate the deviation between actual coordinates and theoretical coordinates (including the tool diameter). Since positive and negative signs will affect the cutting tool direction in the machining process, a vector method that has magnitude and direction is used for conversion. In a cartesian coordinate system, the components of the vector are the projections of the vector along the X and Y direction. After the deviation value was obtained, then the coordinate of the counterpart is corrected according to the deviation value of the part. The counterpart correction coordinate can be calculated by using Equation (6). The shape of the counterpart after compensation is shown in the red solid line in Figure 6b. The assembly matching before and after compensation is shown in Figure 6c,d.

(6) $$\{\begin{array}{c} X_{1}-\Delta_{x}-\frac{1}{2}\emptyset =X_{2}\\ Y_{1}-\Delta_{y}-\frac{1}{2}\emptyset =Y_{2} \end{array}\;$$

where: $X_{1}$, $Y_{1}$ are the actual coordinates of the part; $\Delta_{x}$, $\Delta_{y}$ are error amount *X* and *Y*; ∅ is the cutting tool diameter; $X_{2}$, $Y_{2}$ are the corrected coordinates of the counterpart.

### 2.6. Autogeneration of an Error-Compensated NC Program

To achieve online automatic error compensation, the above-mentioned error conversion results need to be inserted into the NC machining program to correct the cutting tool path. Therefore, it is necessary to declare the processing block for error compensation in the system in advance. In the setting section on the human–machine interface, the key measurement point and the corresponding NC program block is designed. The system will automatically search and identify the set block in the original NC program according to the key measurement setting, then integrate the error compensation amount into the X-, Y-, and Z-coordinate value of the block.

The NC cutting process generally consists of G00, G01, G02, and G03. In this research, Regular Expressions in the C# programming language is used to identify a single block. Regular Expressions is a way to search a pattern with a certain rule string; therefore, this method is suitable for searching NC code in a single block. To automatically generate an error-compensated NC program for a counterpart, the first step is to find the block number to be compensated. After the deviation value is obtained by subtracting the actual coordinate from the theoretical coordinate of the part, then the coordinate of the counterpart is corrected according to the deviation value of the part. The detail of the auto-generated error compensation NC program can be seen in the previous work [1].

The total time for the compensation can be calculated using Equation (7):

(7) $$\mathrm{Total}\;\mathrm{time}=\left(K_{p}\left(\frac{S_{tp}}{V_{tp}}\right)+T_{c}\right)$$

where $K_{p}$ is the number of key measurement points; $V_{tp}$ is the velocity of touchpoint (max 10 mm/s based on Renishaw manual reference); $S_{tp}$ is the distance between the probe and key measurement point (mm); $T_{c}$ is the calculation time of deviation (s).

## 3. Mating Error Compensation System

Based on the proposed error compensation method, an online matching error compensation system for complex shapes was developed in visual C# language. The architecture of the system includes a login system, pre-setting system, and an intelligent error compensation system.

### 3.1. Human–Machine Interface Setup

**Figure 7:**
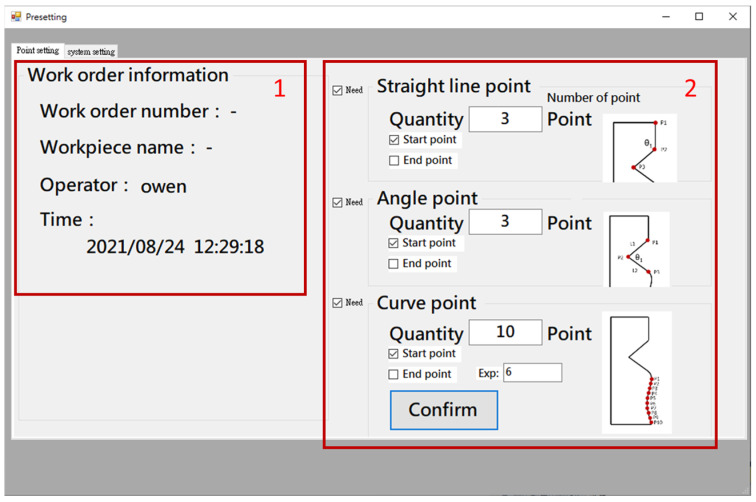
HMI—Point setup interface.

**Figure 8:**
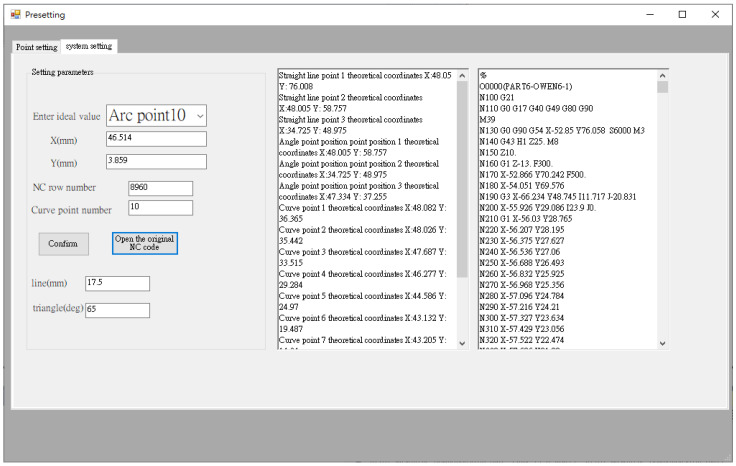
HMI—System setup interface.

Before performing error compensation, the user needs to define the required key point for measurement, and the corresponding NC processing program blocks information for automatic compensation. Figure 7 shows the human–machine interface (HMI) point setup interface. It provides information of work order number, workpiece name, operator, and time in the left area (#1 in Figure 7). On the right side (#2 in Figure 7), the user can enter the number of points to be compensated for straight line, triangle, and curve shapes. The system will record this information and use it for error compensation calculation. After setting the number of points, the next step is to enter the theoretical coordinates of each key point for the straight line, triangle, and curve shapes and the related NC block number, as shown in Figure 8, so that the error can be compensated to the corresponding NC block. Finally, the original NC program of the counterpart is imported.

### 3.2. HMI On-Line Error Compesation

**Figure 9:**
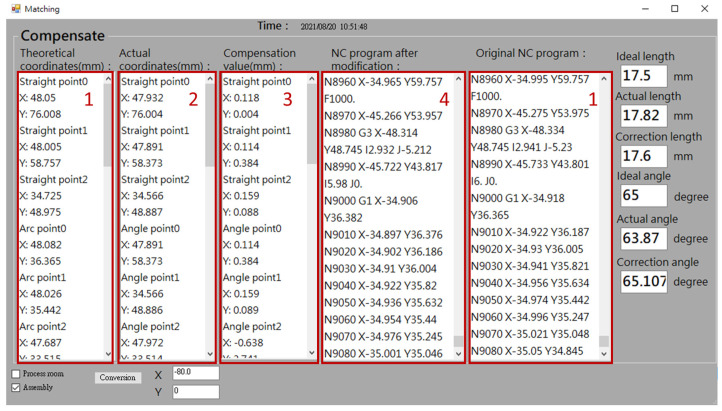
HMI—Error compensation interface.

Figure 9 shows the error compensation interface. The system automatically displays the part and counterpart theoretical coordinates of the key measurement point in the area of the theoretical coordinates and original NC program, respectively (#1 in Figure 9). After the part is measured by the Renishaw Equator 300 instrument, the system will automatically record the actual coordinate measurement value of the part and display it in the actual coordinate area (#2 in Figure 9). Subsequently, the deviation value between theoretical and actual value is calculated, and then displayed in the compensation value area (#3 in Figure 9). Furthermore, the system converts the compensation value into the coordinate of the counterpart and corrects the NC program of the counterpart (#4 in Figure 9). This modified NC program not only can be stored in the edge computer but also automatically uploaded to the CNC controller of the machine tool.

## 4. Experiment and Verification

### 4.1. Experiment Preparation

Experiments were designed to verify the effectiveness of the proposed method for the curve-mating and complex-mating types. Aluminum workpieces were used in the experiments. A Renishaw Equator 300 with a working range of 300 × 300 × 150 mm was used to in-line measure the part. A CNC vertical milling machining center YTM-763 with a travel range of 760 × 400 × 350 mm, maximum spindle speed of 20,000 rpm, and Delta NC300A controller was used to machine the counterpart. A computer with the specification of intel core i7-7700 and RAM 16 GB was used. The cutting parameters for experiment verification are shown in Table 1.

### 4.2. Verification of Curve Mating

**Figure 10:**
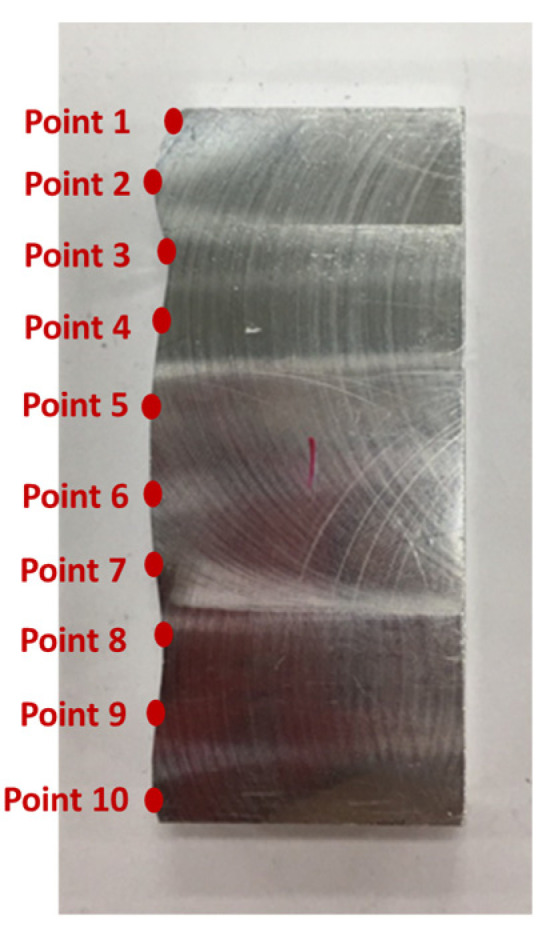
The finished part with curve mating.

**Figure 11:**
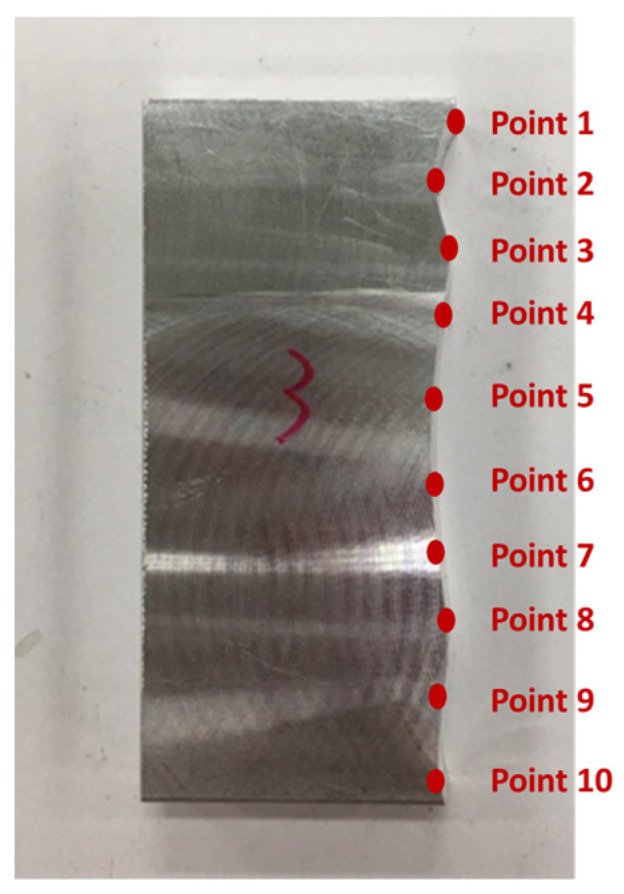
The finished counterpart without compensation.

**Figure 12:**
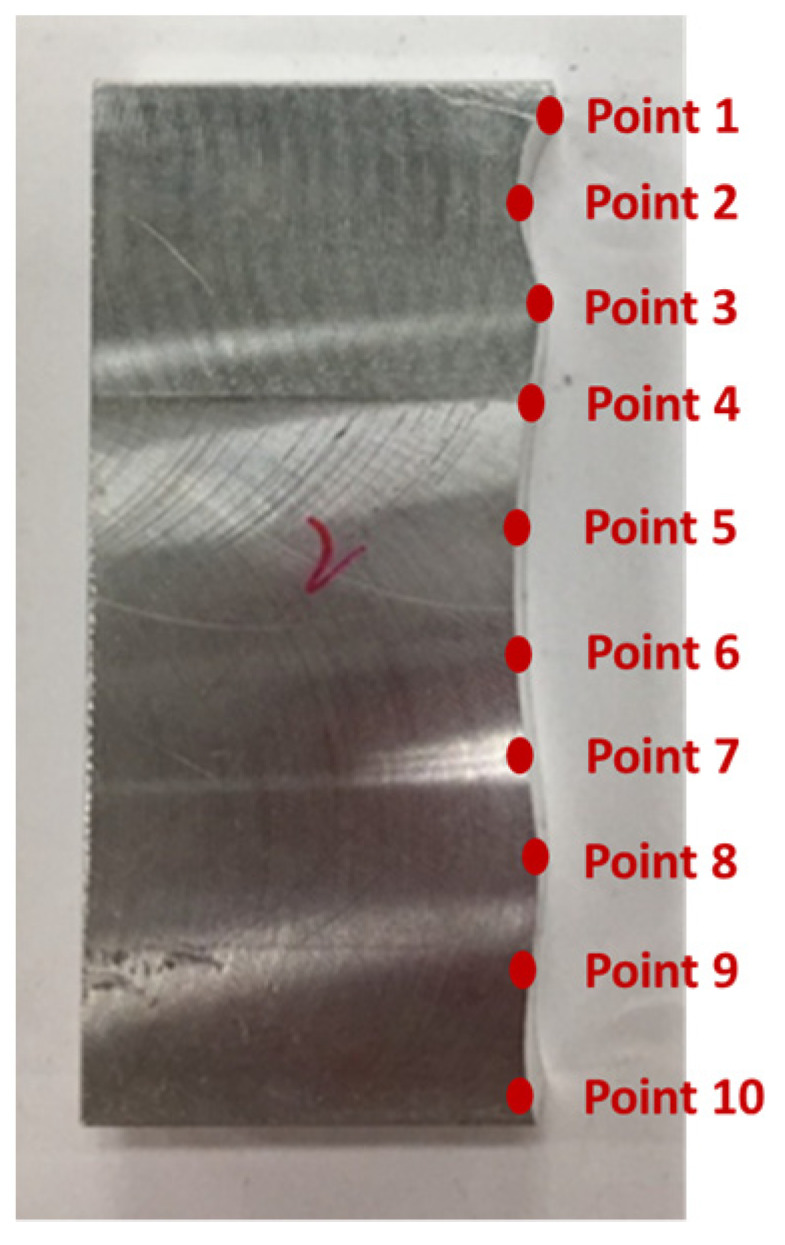
The finished counterpart with compensation.

**Figure 13:**
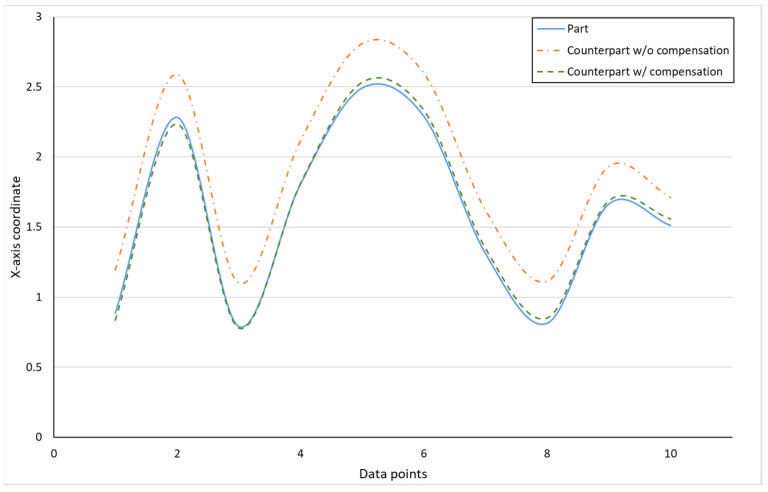
Error comparison between counterpart w/o and w/ compensation for curve mating.

**Figure 14:**
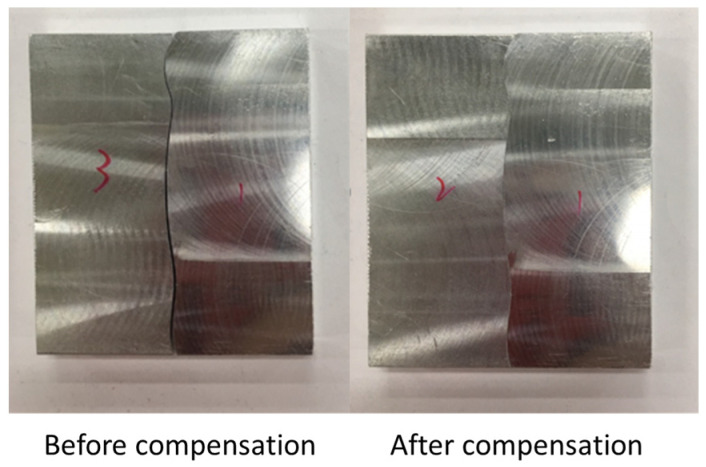
Comparison assembly curve mating before and after compensation.

Figure 10 shows the finished part with curve mating. The cutting parameters used for machining the part are shown in Table 1. The tolerance chain is ±0.05 mm. Ten key points were used in the measurement and compensation. The location of key measurement points are shown in Figure 10, and the corresponding coordinates are shown in Table 2. Figure 11 shows the finished counterpart without compensation and the location of key measurement points. The corresponding measured coordinates and the conversion coordinates are shown in Table 3. Figure 12 shows the finished counterpart with compensation and the location of key measurement points. The corresponding measured coordinates and the conversion coordinates are shown in Table 4. Figure 13 shows the error comparison between counterparts without and with compensation. It can be seen that the largest error for counterpart without compensation is 0.116 mm, which exceeds the tolerance range of 0.05 mm. After compensation, the error becomes 0.048 mm, which is within the tolerance range. Consequently, the assembly accuracy increase to 28%. Figure 14 shows the comparison assembly matching before and after compensation. It can be seen that there is a quite big gap in between part and counterpart for the assembly matching before compensation. On the contrary, the mating in the assembly matching after compensation exhibits good matching.

### 4.3. Verification of Complex Mating

**Figure 15:**
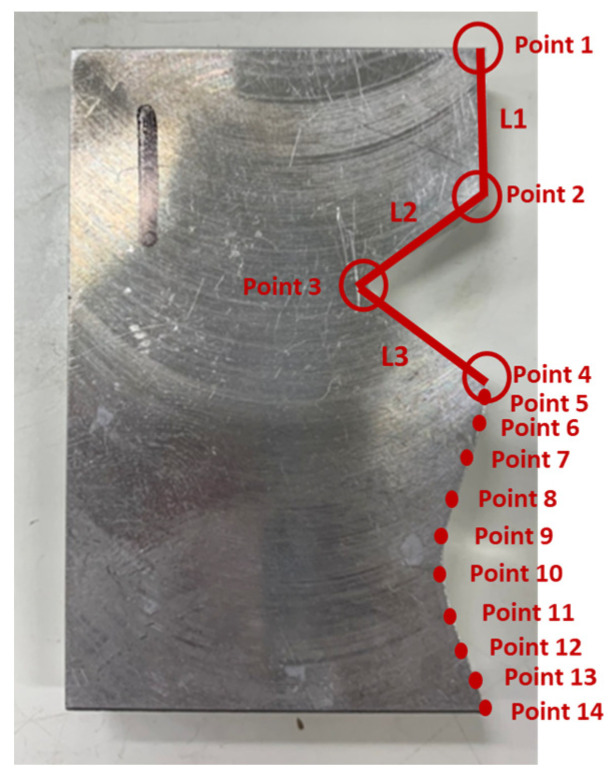
The finished complex part.

**Figure 16:**
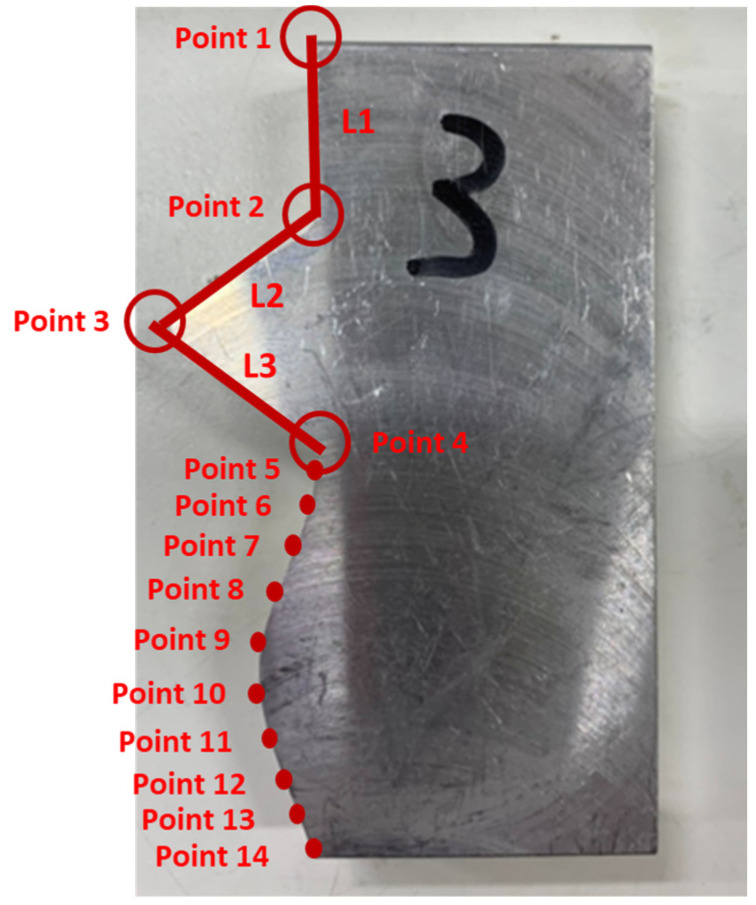
The finished complex counterpart without compensation.

**Figure 17:**
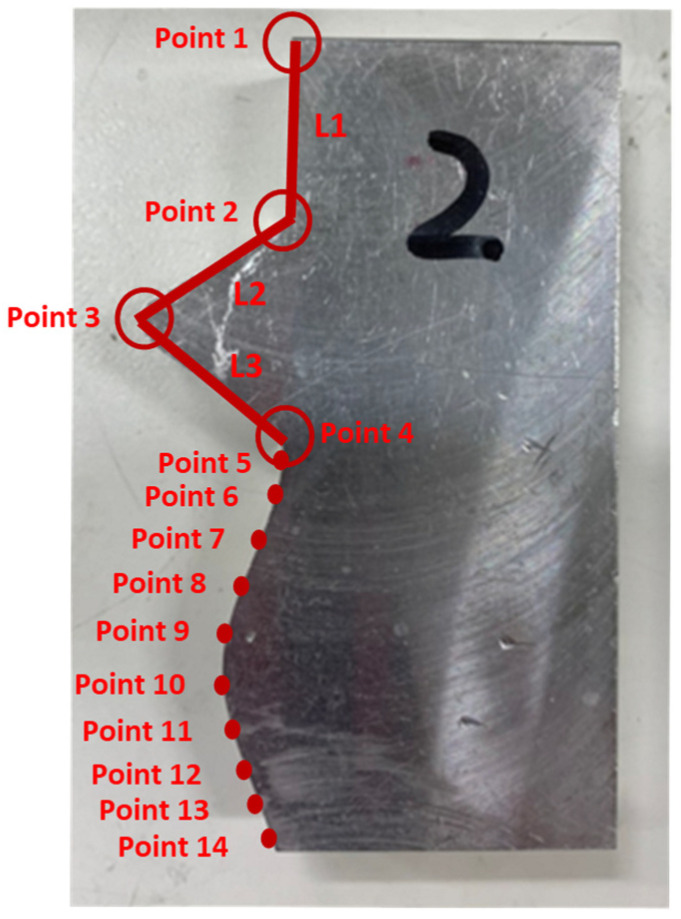
The finished complex counterpart with compensation.

The verification for complex mating consists of straight line, triangle, and curve shapes, as shown in Figure 15. The straight line part is represented by L1, whereas the triangle shape is represented by the combination of L2, L3, and the angle in between. Meanwhile, the curve shape is represented by point 5 to point 14. The corresponding measured coordinates of the complex part were shown in Table 5. The cutting parameters for this experiment were shown in Table 1. Figure 16 showed the finished complex counterpart without compensation and the location of key measurement points. The corresponding measured coordinates and the conversion coordinates were shown in Table 6. Figure 17 showed the finished complex counterpart with compensation and the location of key measurement points. The corresponding measured coordinates and the conversion coordinates were shown in Table 7.

The actual distance between point 1 and point 2 of the straight line shape of the complex part was 17.649 mm, and the angle between L1 and L2 was 118.948°. Before compensation, the actual distance between point 1 and point 2 of the complex counterpart was 17.308 mm, thus the deviation was 0.341 mm. Meanwhile, the angle between L1 and L2 was 119.542°, thus the deviation was 0.594°. After compensation, the actual distance between point 1 and point 2 of the complex counterpart was 17.592 mm, which indicated that the error decreased from 0.341 mm to become 0.057 mm. Furthermore, the actual angle between L1 and L2 was 119.061°, which demonstrated an error reduction of 81%.

In the triangle shape of the complex part, the actual angle between L2 and L3 was 63.948°. Before the compensation, the angle between L2 and L3 of the complex counterpart was 64.878°, which indicated an error of 0.93°. After the compensation, the angle between L2 and L3 of the complex counterpart was 63.83°, which showed that the error was reduced to 0.118°, and, therefore, an improvement of 87% as a consequence.

**Figure 18:**
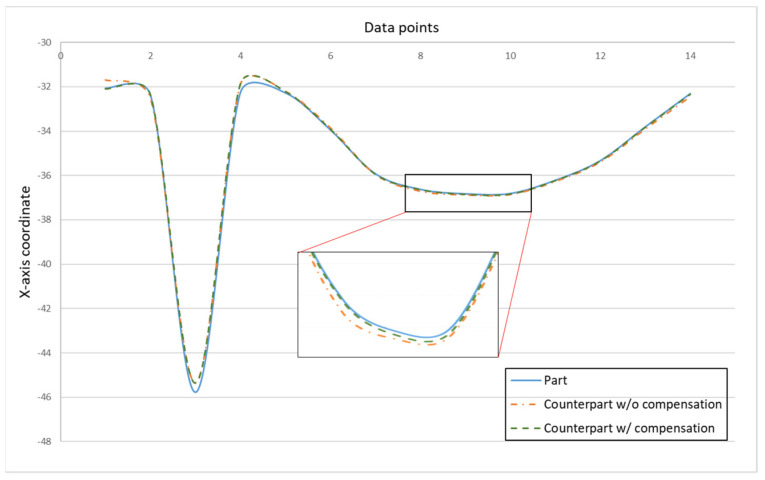
Error comparison between counterpart w/o compensation vs. counterpart w/ compensation for complex mating.

**Figure 19:**
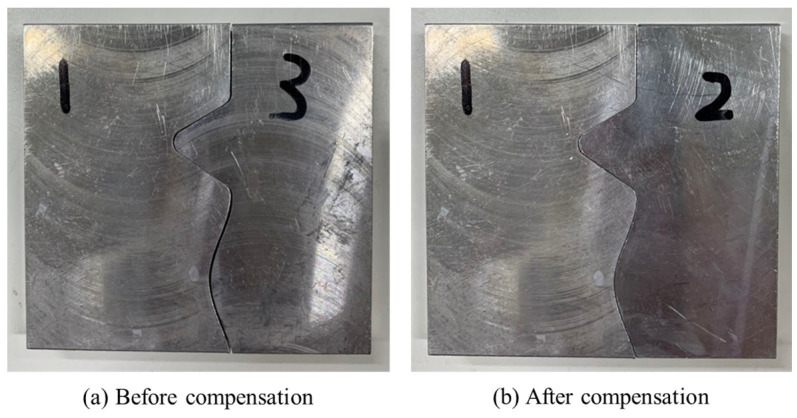
Comparison assembly complex mating before and after compensation.

Figure 18 showed the error comparison between counterparts without and with compensation for a complex part. It can be seen for the curved shape of the complex part that the error before compensation was 0.137 mm, whereas the error after compensation was reduced to 0.029 mm, thus the accuracy improved by 79% as a consequence. However, a large error has occurred in two areas. The first area is the meeting point between curve and triangle that showed errors of 0.371 mm and 0.391 mm before and after compensation, respectively. This is because the corner of the meeting point cannot be chosen as the key measurement point; therefore, only the nearest to the corner can be chosen, which leads to a larger error. The second area is the inside corner of the triangle. The inside corner of a triangle cannot be reached by the probe of the Renishaw Equator due to the ball shape of the probe with a certain radius. In addition to the probe ball shape reason, the polynomial curve fitting method used to reconstruct the shape may result in overfitting due to higher order polynomials. Nevertheless, the majority of errors were small and in the tolerance range after compensation. Figure 19 showed the comparison assembly of complex mating before and after compensation. It can be seen, before compensation, that the gap in between the complex part and its counterpart is quite large due to the larger error. Meanwhile, the gap in between the complex part and its counterpart after compensation was smaller and exhibited better matching.

The total time for this complex shape compensation can be calculated by using Equation (7). The given parameters data: key measurement points were 14, the velocity of touchpoint was 10 mm/s, the distance between the probe and key measurement point was 5 mm, and the calculation time of deviation was 1 s. After substituting these values into Equation (7), it showed that the total time for this complex shape compensation was 8 s.

## 5. Conclusions

In this study, an error-matching compensation method for curve mating and complex mating was developed. Error analysis and algorithms for error identification and error compensation of curve mating and complex mating were proposed. Polynomial curve fitting method and least squares method were used for error analysis. Based on the proposed method, an online error matching compensation system was built. Experiment verification results showed that the proposed method can improve the precision of assembly matching. For curve mating, the matching error before compensation was 0.116 mm, but the error decrease becomes 0.048 mm after compensation so that the assembly accuracy increase to 28% as consequence. For complex mating, the experiment verification results showed error reduction after compensation for each shape, which are 81%, 87%, and 79% for straight line, triangle, and curve shape, respectively. Accordingly, the precision assembly of complex mating will also be improved.

## Figures and Tables

**Table 1:** Cutting parameters for experiment verification.

Subject	Curve	Complex
Tool diameter (mm)	6	6
Spindle speed (rpm)	6000	6000
Feed rate (mm/min)	500	500
Material	Aluminum	Aluminum
Tool material	Tungsten Carbide	Tungsten Carbide
Tool flutes	3	3
Width of Cut (mm)	0.1	0.2
Depth of Cut (mm)	13.5	13.5

**Table 2:** The measured coordinates of the part.

Point	X (mm)	Y (mm)
1	0.883	−2.819
2	2.281	−8.381
3	0.790	−15.772
4	1.803	−25.847
5	2.490	−35.824
6	2.290	−43.426
7	1.311	−51.845
8	0.812	−61.008
9	1.663	−68.944
10	1.510	−73.454

**Table 3:** The measured coordinates and conversion coordinates of counterpart without compensation.

Point	Measured Coordinate		Converted Coordinate	
	X (mm)	Y (mm)	X (mm)	Y (mm)
1	1.193	−2.818	−2.006	−2.768
2	2.591	−8.382	−0.655	−8.332
3	1.100	−15.769	−2.176	−15.719
4	2.113	−25.866	−1.191	−25.816
5	2.810	−35.823	−0.542	−35.773
6	2.600	−43.425	−0.766	−43.375
7	1.621	−51.843	−1.799	−51.793
8	1.112	−61.007	−2.304	−60.957
9	1.933	−68.943	−1.445	−68.893
10	1.710	−73.453	−1.567	−73.403

**Table 4:** The measured coordinates and conversion coordinates of counterpart with compensation.

Point	Measured Coordinate		Converted Coordinate	
	X (mm)	Y (mm)	X (mm)	Y (mm)
1	0.835	−2.818	−2.217	−2.719
2	2.236	−8.380	−0.964	−8.383
3	0.779	−15.769	−2.421	−15.779
4	1.808	−25.846	−1.181	−25.843
5	2.533	−35.822	−0.377	−35.824
6	2.335	−43.425	−0.535	−43.426
7	1.439	−51.843	−1.361	−51.847
8	0.851	−61.007	−1.751	−61.008
9	1.688	−68.943	−0.808	−68.944
10	1.558	−73.452	−0.995	−73.468

**Table 5:** The measured coordinates of complex part.

Point	X (mm)	Y (mm)	Angle (Deg)	Distance (mm)
1	−32.067	76.005		17.649
2	−32.372	58.359	118.948	
3	−45.769	49.020	63.948	
4	−32.266	37.782		
5	−32.280	33.047		
6	−33.943	28.052		
7	−35.924	22.701		
8	−36.626	19.804		
9	−36.827	17.891		
10	−36.812	16.453		
11	−36.217	12.695		
12	−35.332	9.749		
13	−33.811	5.734		
14	−32.311	1.794		

**Table 6:** The measured coordinates and conversion coordinates of complex counterpart without compensation.

Point	Measured Coordinates				Converted Coordinates			
	X (mm)	Y (mm)	Angle (Deg)	Distance (mm)	X (mm)	Y (mm)	Angle (Deg)	Distance (mm)
1	−31.691	76.003		17.308	−32.443	76.003		17.308
2	−32.524	58.715	119.542		−32.220	58.715	119.542	
3	−45.345	49.027	64.878		−46.193	49.027	64.878	
4	−31.895	37.792			−32.637	37.792		
5	−32.206	33.064			−32.354	33.064		
6	−33.853	28.066			−34.033	28.066		
7	−35.955	22.716			−35.893	22.716		
8	−36.712	19.803			−36.540	19.803		
9	−36.878	17.888			−36.776	17.888		
10	−36.862	16.455			−36.762	16.455		
11	−36.27	12.696			−36.164	12.696		
12	−35.396	9.746			−35.268	9.746		
13	−33.907	5.733			−33.715	5.733		
14	−32.448	1.792			−34.033	1.792		

**Table 7:** The measured coordinates and conversion coordinates of complex counterpart with compensation.

Point	Measured Coordinates				Converted Coordinates			
	X (mm)	Y (mm)	Angle (Deg)	Distance (mm)	X (mm)	Y (mm)	Angle (Deg)	Distance (mm)
1	−32.101	76.006		17.592	−32.033	76.006		17.562
2	−32.367	58.446	119.061		−32.377	58.446	119.061	
3	−45.365	49.027	63.83		−46.173	49.027	63.83	
4	−31.875	37.792			−32.657	37.792		
5	−32.224	33.054			−32.336	33.064		
6	−33.918	28.066			−33.968	28.066		
7	−35.947	22.716			−35.901	22.716		
8	−36.64	19.803			−36.612	19.803		
9	−36.853	17.888			−36.801	17.888		
10	−36.841	16.455			−36.783	16.455		
11	−36.234	12.696			−36.200	12.696		
12	−35.353	9.746			−35.311	9.746		
13	−33.827	5.731			−33.795	5.733		
14	−32.34	1.791			−32.282	1.792		
